# Mitochondrial Dysfunction at the Crossroads of Necroptosis: Mechanisms, Molecular Mediators, and Therapeutic Opportunities

**DOI:** 10.1111/jcmm.71252

**Published:** 2026-07-28

**Authors:** Fengxin Wang, Haiying Rui, Dandan Qin, Huaxiang Yu, Dan Zou, Wenyi Zou, Yuting Li, Ran Li, Yuguo Chen, Li Xue

**Affiliations:** ^1^ Department of Emergency Medicine Qilu Hospital of Shandong University Jinan China; ^2^ Shandong Provincial Clinical Research Center for Emergency and Critical Care Medicine, Institute of Emergency and Critical Care Medicine of Shandong University, Chest Pain Center Qilu Hospital of Shandong University Jinan China; ^3^ Key Laboratory of Emergency and Critical Care Medicine of Shandong Province, Key Laboratory of Cardiopulmonary‐Cerebral Resuscitation Research of Shandong Province, Shandong Provincial Engineering Laboratory for Emergency and Critical Care Medicine Qilu Hospital of Shandong University Jinan China; ^4^ Shandong Key Laboratory, Magnetic Field‐Free Medicine & Functional Imaging (MF), Qilu Hospital of Shandong University Jinan China; ^5^ NMPA Key Laboratory for Clinical Research and Evaluation of Innovative Drug Qilu Hospital of Shandong University Jinan China

**Keywords:** cancer therapy, cardiovascular disease, inflammatory pathways, mitochondrial dysfunction, necroptosis, neurodegeneration

## Abstract

The conceptual landscape of cell death has evolved beyond the traditional dichotomy of apoptosis and necrosis to encompass diverse regulated pathways including necroptosis, autophagy, ferroptosis, and pyroptosis. Necroptosis, a caspase‐independent inflammatory form of programmed cell death, has emerged as a critical driver of the pathogenesis of cardiovascular disorders, neurodegenerative diseases, and cancer. Concurrently, our understanding of mitochondrial biology has undergone a paradigm shift: mitochondria are no longer viewed merely as bioenergetic powerhouses, but as dynamic signalling hubs that orchestrate metabolic reprogramming, cellular homeostasis, and ultimate cell fate decisions. In this regard, a growing body of evidence suggests that mitochondrial dysfunction is a central rheostat that enables necroptotic execution. This review delineates the mechanistic interplay between necroptosis and mitochondrial dysfunction and systematically analyzes the key molecular mediators and pathological pathways through which mitochondrial dysregulation drives necroptotic activation. Furthermore, this review identifies actionable therapeutic targets and translational strategies for modulating necroptosis in related diseases.

## 1 Introduction

Mitochondria, traditionally recognized as the cellular powerhouses for ATP synthesis via oxidative phosphorylation, are now appreciated as multifunctional signalling hubs that orchestrate metabolism, redox homeostasis, and cell fate decisions [1]. Their functionality depends on their structural integrity and dynamic regulatory mechanisms, including mitochondrial quality control (MQC) systems, ion homeostasis, and regulated opening of the mitochondrial permeability transition pore (mPTP). Mitochondria act as sentinel organelles that detect cellular stress and integrate signals across programmed cell death pathways, including apoptosis, necroptosis, pyroptosis, ferroptosis, and non‐programmed necrosis. Necroptosis is a receptor‐interacting protein kinase 1 and 3 (RIPK1/RIPK3)/mixed lineage kinase domain‐like pseudokinase (MLKL)‐driven inflammatory cell death modality [2, 3]. Morphologically, necroptosis is characterized by cell swelling, plasma membrane rupture, mitochondrial cristae disorganization, matrix swelling, and organelle fragmentation [4]. Mounting evidence suggests that mitochondrial dysfunction can modulate or amplify necroptotic signalling in a context‐dependent manner. Disruption of MQC (e.g., impaired mitophagy and proteostatic collapse), bioenergetic failure (reduced ATP synthesis), and mPTP dysregulation converge to induce mitochondrial demise. These insults manifest as structural aberrations—cristae disassembly, matrix swelling, and mitochondrial fragmentation—accompanied by reactive oxygen species (ROS) bursts, membrane potential (ΔΨm) dissipation, and damage‐associated molecular pattern (DAMP) release (e.g., mitochondrial DNA, cytochrome c). Such events activate necrosome assembly, bridging mitochondrial dysfunction and inflammatory tissue damage in diseases ranging from ischemia–reperfusion injury to neurodegeneration.

RIPK1/RIPK3/MLKL cascade activation is a central event in necroptosis execution; nonetheless, its interplay with mitochondrial dysfunction remains incompletely understood. Emerging evidence suggests that the phosphorylation‐driven activation of RIPK1/RIPK3 is critical for necroptosis progression, revealing a complex bidirectional regulatory interplay with mitochondrial structural destabilization. Current understanding suggests that mitochondrial dysfunction operates through a self‐amplifying cycle, acting as an initiator that triggers RIPK1‐RIPK3 phosphorylation cascades, and as a consequential effector of these activated pathways. This reciprocal relationship creates a pathogenic feed‐forward loop that drives necroptotic cell death. Consequently, evaluating the precise temporal sequence of mitochondrial events following necroptosis initiation, particularly the key molecular mediators governing mitochondria‐kinase crosstalk, represents a crucial frontier in decoding the full pathomechanistic circuitry of necroptosis. Such insights may reveal novel therapeutic targets for modulating the lytic cell death pathway in various diseases.

In this review, we systematically dissect the multifactorial drivers of mitochondrial structural and functional perturbations, with a dedicated focus on MQC systems, bioenergetic dysregulation, and mPTP abnormalities, which are critical axes governed by distinct molecular effectors. These insights establish a conceptual framework for prioritising mitochondria‐centric therapeutic strategies while charting new frontiers for probing the spatiotemporal regulation of necroptosis.

## 2 The Mitochondrial Quality Control System and Necroptosis

Under pathological stress, the MQC operates as a dynamic triage system that orchestrates fission/fusion balance, mitophagy, biogenesis, and proteostasis to safeguard mitochondrial integrity [5]. Paradoxically, under specific pathological conditions, failure of certain MQC axes may convert this surveillance network into a necroptotic trigger: (1) dysregulated fission‐fusion dynamics that fragment mitochondrial networks; (2) defective mitophagy, which permits the accumulation of damaged organelles; (3) impaired biogenesis, which depletes functional mitochondrial reserves; and (4) collapsed protein homeostasis, inducing proteotoxic stress. These intersecting failures synergistically licence necroptosis by disrupting metabolic flux, amplifying ROS‐RIPK1/3 crosstalk, and releasing mitochondrial DAMPs.

### 2.1 Mitochondrial Dynamics

#### 2.1.1 Mitochondrial Fission

Mitochondrial fission is a dynamic process essential for organelle quality control and is governed by the precise regulation of dynamin‐related protein 1 (Drp1). This cytosolic GTPase undergoes post‐translational modifications at conserved serine residues S616 and S637. Specifically, phosphorylation at S616 drives Drp1 recruitment to the mitochondrial outer membrane (OMM) to execute fission, while phosphorylation at S637 classically inhibits fission by retaining Drp1 in the cytoplasm [6]. However, emerging evidence suggests that S637 dephosphorylation synergizes with S616 activation under pathological conditions [7]. This dual regulatory mechanism suggests that Drp1 functions as a molecular rheostat that integrates stress signals to determine mitochondrial morphology and cellular fate.

Phosphoglycerate mutase 5 (PGAM5), an OMM‐localized phosphatase and RIPK3 substrate, orchestrates Drp1 activation through dual mechanisms: dephosphorylating Drp1 S637 to release its autoinhibitory conformation and facilitating S616 phosphorylation via unidentified kinases [8]. Mechanistic studies across diverse disease models have consistently implicated the PGAM5‐Drp1 axis as a central executor of mitochondrial fission‐linked necroptosis. Wang et al. demonstrated that RIPK1/RIPK3/MLKL necrosome assembly precedes PGAM5‐mediated recruitment and S637 dephosphorylation of Drp1 in TNF‐α‐stimulated HeLa cells, triggering hallmark mitochondrial fragmentation as an early necroptotic checkpoint [9]. A complementary study by Zhu et al. revealed that PGAM5‐dependent Drp1 S637 dephosphorylation in myocardial injury models was correlated with mitochondrial membrane potential (MMP) dissipation, ATP depletion, and mitochondrial ROS (mtROS) amplification [10]. In contrast, Li et al. showed that antioxidant pretreatment (NAC/APP) in mesenchymal stem cells suppressed Drp1 S616 phosphorylation and mitochondrial clustering, rescuing cells from necroptosis [11]. Notably, Luo et al. extended this paradigm to neuropathology, demonstrating that TNF‐α‐driven RIPK1/RIPK3/PGAM5 signalling induces oligodendrocyte necroptosis via Drp1 S616 phosphorylation and optic atrophy 1 (OPA1) downregulation in multiple sclerosis models (EAE and cuprizone‐induced demyelination) [12]. Parallel findings in ovarian aging by Li et al. revealed age‐associated PGAM5 accumulation in granulosa cells, which scaffolds Drp1 oligomerization at the mitochondria, elevates S616 phosphorylation (2.4‐fold), and reduces mitochondrial length by 62%, thereby promoting necroptosis [13]. Collectively, these studies identify PGAM5 as a bifunctional regulator of Drp1 activation, orchestrating S637 dephosphorylation and S616 phosphorylation to drive mitochondrial fission and necroptosis in multiple cellular stress contexts. Nevertheless, the extent to which this mechanism applies universally across different disease models and stimuli warrants further investigation.

**FIGURE 1:**
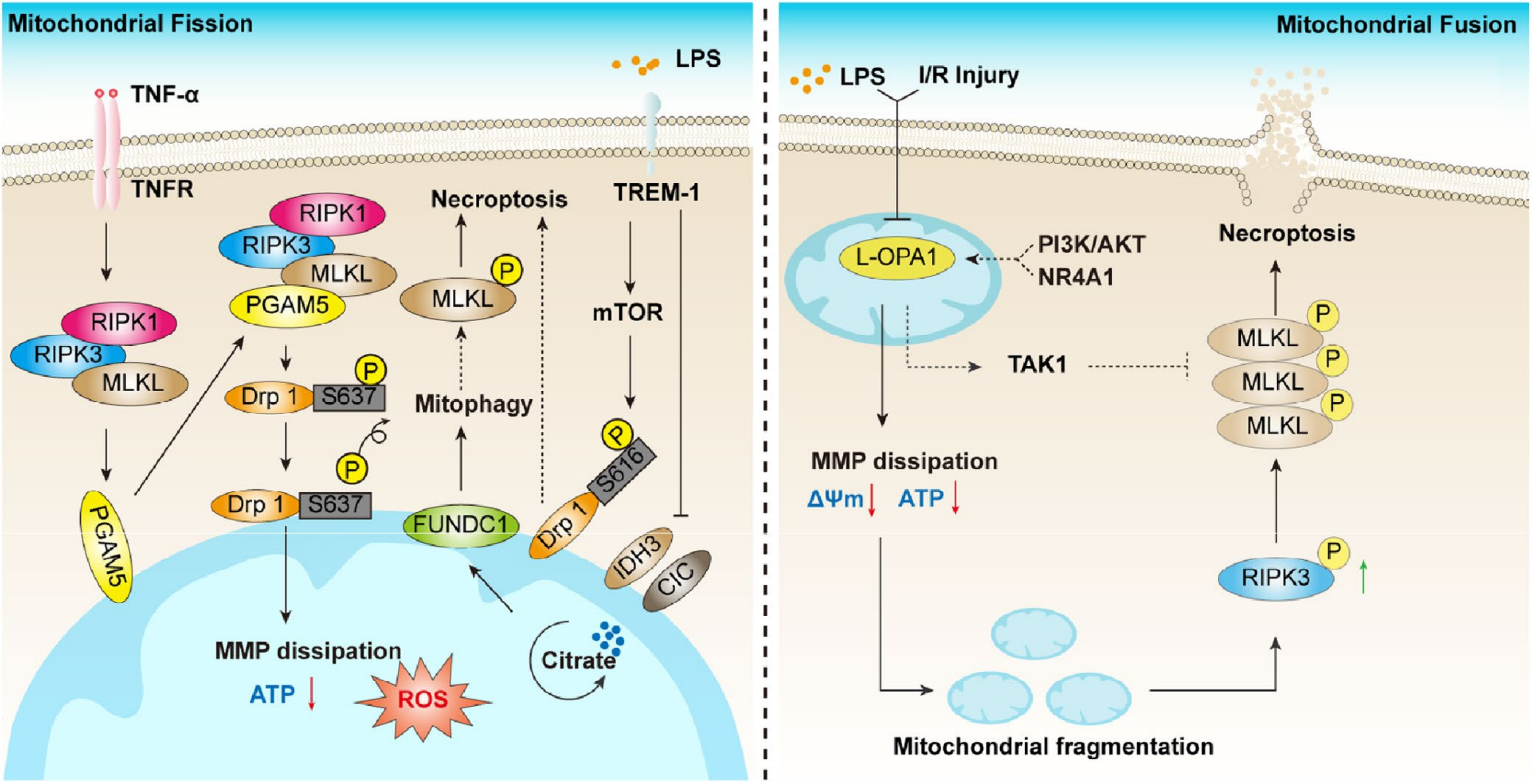
Mechanism of mitochondrial dynamics in the development of necroptosis. Mitochondrial fission‐driven necroptosis is mainly regulated by Drp1 phosphorylation. Phosphorylation of Drp1 at S616 drives its fission at the outer mitochondrial membrane, while dephosphorylation at S637 (mediated by PGAM5) disinhibits it and synergistically promotes S616 phosphorylation. As a bifunctional factor, PGAM5 fully activates Drp1 through S637 dephosphorylation and promotes S616 phosphorylation. In addition, the TREM‐1/mTOR pathway directly phosphorylates S616, whereas IDH3α/CIC inhibition indirectly triggers necrosis via FUNDC1‐mediated mitophagy. Excessive fission leads to membrane potential collapse, ATP depletion, and a burst of reactive oxygen species, ultimately triggering necroptosis. Conversely, mitochondrial fusion triggers necroptosis through the following molecular mechanisms: Stress induces OMA1/YME1L proteases to cleave OPA1, reducing the long isoform L‐OPA1, leading to mitochondrial fragmentation, loss of ΔΨm, and functional collapse. This disruption promotes activation of the RIPK1/RIPK3 kinase complex and phosphorylation of MLKL. Upstream PI3K/AKT, NR4A1 regulates OPA1 expression, while reduced downstream TAK1 (RIPK1 inhibitor) expression (negatively correlated with OPA1 levels) further amplifies the necrotic signal.

However, Drp1 regulation exhibits striking heterogeneity across various pathological contexts. Zhong et al. identified TREM‐1/mTOR‐mediated S616 phosphorylation driving alveolar macrophage necroptosis via MMP collapse [14], while Yang et al. revealed that IDH3α/CIC suppression‐induced citrate accumulation in epithelial cells hyperactivates FUN14 Domain Containing 1 (FUNDC1)‐dependent mitophagy, indirectly triggering MLKL phosphorylation in acute lung injury [15]. PGAM5 acts as a central bifunctional regulator of Drp1 (dephosphorylating S637 and facilitating S616 phosphorylation) to drive mitochondrial fission and necroptosis across diverse disease contexts, although alternative Drp1 activation pathways exist in specific pathologies (Figure 1).

Although most studies support Drp1‐mediated fission as a pro‐necroptotic mechanism, some studies have reported contradictory evidence. Maeda et al. observed enhanced TNF‐α‐induced necroptosis in Drp1‐inhibited (Mdivi‐1‐treated) Fas‐associated death domain (FADD)‐deficient Jurkat cells [16], whereas Remijsen et al. reported delayed necroptosis only in PGAM5‐knockdown mice [17]. Species‐specific effects further complicate this paradigm, with PGAM5 inhibition exacerbating cardiac injury in rats but attenuating it in mice (Figure 1).

Consequently, a multidimensional framework combining structural metrics, functional assays, and dynamic live‐cell imaging of Drp1 translocation and RIPK3‐PGAM5 interactions is critical. This approach distinguishes the primary drivers of mitochondrial collapse from secondary effects. Additionally, targeting the PGAM5‐Drp1 axis offers two strategies: pharmacological inhibitors to block fission in acute conditions, such as I/R injury, and CRISPR‐mediated editing of Drp1 phosphorylation sites for precise regulation.

#### 2.1.2 Mitochondrial Fusion

Mitochondrial fusion, a dynamic process essential for organelle homeostasis, involves the coordinated merging of two membranes: outer membrane fusion mediated by mitofusin 1/2 (MFN1/2) and inner membrane fusion orchestrated by OPA1 [18]. This fusion mechanism enables functional complementation between mitochondria, redistribution of metabolites, and DNA to counteract age‐related functional decline. However, the dysregulation of fusion proteins triggers catastrophic consequences. MFN1/2 or OPA1 deficiencies can disrupt mitochondrial networks, potentially leading to bioenergetic collapse, ROS overload, and in certain contexts, necroptosis. Moreover, emerging evidence links these fusion defects to disease progression in neurodegeneration, I/R injury, and metabolic disorders, where fragmented mitochondria serve as biomarkers and mediators of inflammatory cell death. Notably, the balance between OPA1 isoforms acts as a rheostat for cell fate decisions. Long isoforms (L‐OPA1) maintain cristae architecture and fusion competence, whereas stress‐induced cleavage to short isoforms (S‐OPA1) by the protease OMA1/YME1L initiates mitochondrial fragmentation, creating a permissive environment for RIPK1/3 activation [19].

Accordingly, the proteolytic processing of OPA1 has emerged as a pivotal checkpoint in necroptotic signalling. Studies across various disease models have revealed that L‐OPA1 depletion correlates with necroptosis exacerbation. Jiang et al. demonstrated that pharmacological L‐OPA1 inhibition amplified mitochondrial fragmentation, ΔΨm loss, and MLKL phosphorylation in alveolar epithelial cells during acute lung injury, worsening necroptosis [20]. Conversely, Sun et al. showed that restoring L‐OPA1 (via Opa1‐ΔS1 mutants) preserved ATP synthesis and mitochondrial networks, attenuating RIPK3‐dependent neuronal death in retinal I/R [21]. Parkinson's disease models further implicate OPA1 cleavage in dopaminergic neuron necroptosis via DRP1‐MLKL crosstalk [22]. Although these findings suggest that OPA1 is a necroptotic gatekeeper, critical knowledge gaps persist. For instance, upstream regulators such as the PI3K/AKT pathway and NR4A1 transiently modulate OPA1 expression, whereas downstream effectors involve TAK1, a RIPK1 inhibitor whose expression inversely correlates with OPA1 levels [23, 24]. Future research must delineate spatiotemporal OPA1‐kinase interactions and develop isoform‐specific modulators to harness fusion‐necroptosis crosstalk therapeutically (Figure 1).

### 2.2 Mitophagy

#### 2.2.1 Ub‐Dependent Pathways

The Ub‐dependent mitophagy pathway is predominantly governed by the PINK1/Parkin axis and initiates mitochondrial depolarization [25]. Under physiological conditions, PINK1 is rapidly imported into the inner mitochondrial membrane (IMM) and degraded [26]. However, mitochondrial damage stabilizes PINK1 in the OMM, where it phosphorylates Ub at Ser65. Subsequently, PINK1 recruits and activates Parkin, an E3 Ub ligase that ubiquitinates OMM proteins, marking damaged mitochondria for autophagic clearance [27]. This tightly regulated process ensures MQC by selectively removing dysfunctional organelles.

Proper PINK1/Parkin‐mediated mitophagy antagonizes necroptosis by eliminating ROS‐producing mitochondria and preventing the release of DAMPs. For example, PGAM5 stabilizes PINK1 to enhance mitophagy and reduce ROS production and necroptosis in cardiac I/R models [28]. Similarly, tetramethylpyrazine activates PINK1/Parkin‐mediated mitophagy by restoring ubiquinone‐cytochrome c reductase core protein 2 (UQCRC2), mitigating alcohol‐induced hepatocyte necroptosis [29]. Mitophagy activation is temporally correlated with RIPK1/MLKL downregulation in *Staphylococcus aureus* pneumonia, suggesting a protective role [30]. Additionally, Xu et al. provided mechanistic insights using a rat spinal cord injury (SCI) model and oxygen–glucose deprivation (OGD)‐treated PC12 cells, showing that Schwann cells activate mitochondrial AMPK phosphorylation to amplify PINK1/Parkin‐mediated mitophagy, thereby reducing RIPK1/RIPK3/MLKL phosphorylation cascades and necroptosis [31]. Together, these studies highlight the ability of mitophagy to suppress necroptosis by maintaining mitochondrial homeostasis.

**FIGURE 2:**
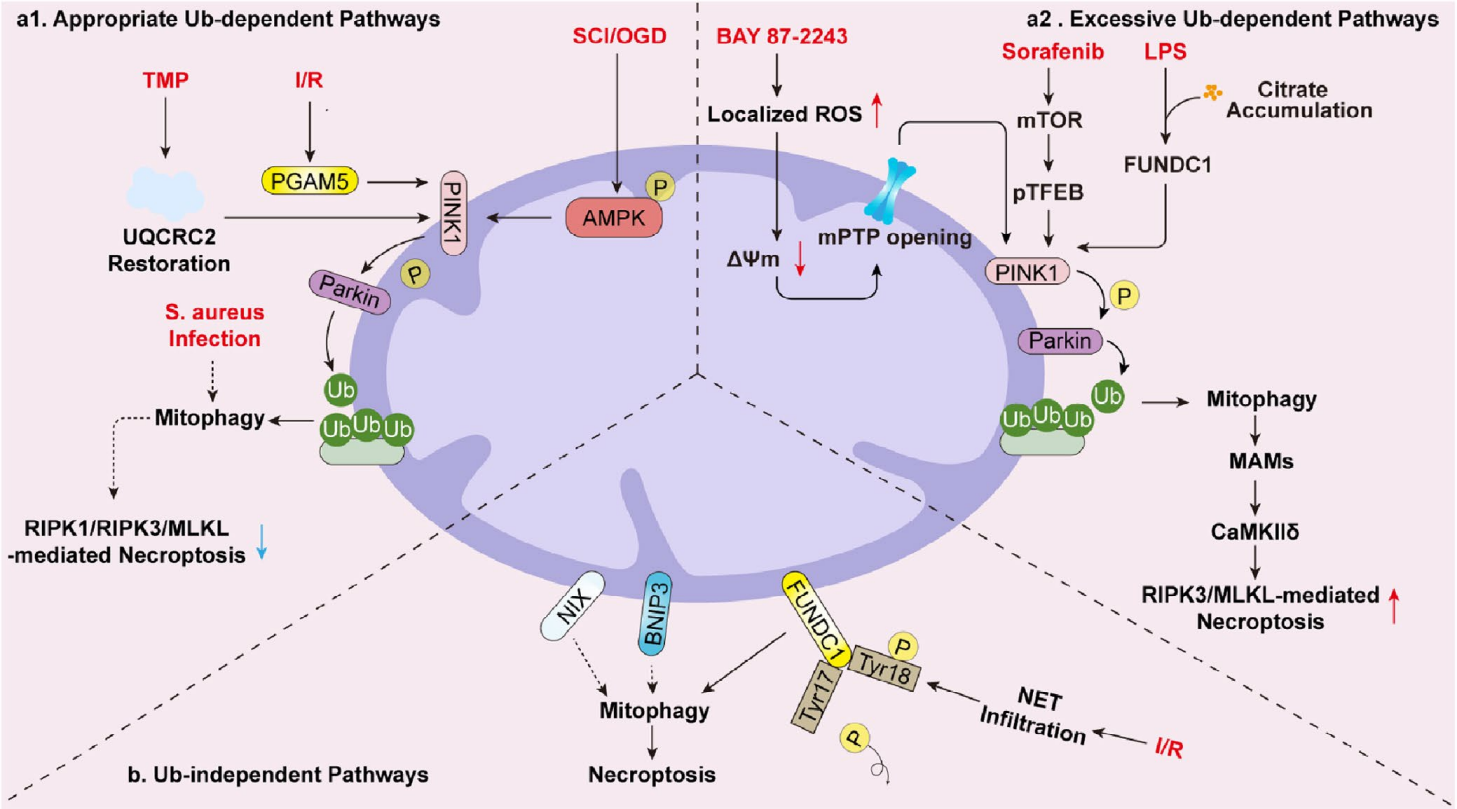
Mechanisms of mitophagy in causing necroptosis. In an appropriate Ub‐dependent pathway, physiological PINK1/Parkin mediated mitophagy antagonises the RIPK1/RIPK3/MLKL necrosis cascade by removing damaged mitochondria, inhibiting ROS production and DAMP release. This process involves activation of the PINK1/Parkin axis by AMPK phosphorylation and stimuli include I/R, SCI/OGD, TMP, and *S. aureus* infection. Excessive Ub‐dependent pathway activation leads to ROS accumulation, MFN2 degradation and mitochondrial‐endoplasmic reticulum contacts destruction through dysregulated PINK1/Parkin autophagy and activates CaMKIIδ‐RIP3/MLKL pathway to aggravate necrosis. The key molecules, including mTOR‐pTFEB, drive Parkin activation and FUNDC1 co‐cleavage. In the Ub‐independent pathway, NIX/BNIP3L, BNIP3, and FUNDC1 receptors directly bind LC3 via LIRs to clear mitochondria and maintain homeostasis to inhibit necroptosis. However, when FUNDC1 phosphorylation is disturbed by NET infiltration (such as by Tyr18 elevation) during I/R stimulation, NIX/BNIP3L, BNIP3, and FUNDC1 receptors maintain homeostasis to inhibit necroptosis. This can impair autophagy and promote necroptosis.

Excessive or dysregulated mitophagy can exacerbate necroptosis. BAY 87–2243, a mitochondrial oxidative phosphorylation (OXPHOS) inhibitor, induces localized ROS accumulation, mitochondrial depolarization, and mPTP opening in BRAFV600E melanoma cells [32]. This cascade subsequently triggers ATG5‐dependent autophagosome formation and aberrant PINK1‐mediated mitophagy, which further amplifies ROS production and ultimately promotes necrosome assembly and necroptosis. Similarly, sorafenib activates PINK1/Parkin via mTOR‐pTFEB, degrading MFN2 and disrupting mitochondrial‐endoplasmic reticulum (ER) contacts, thereby activating CaMKIIδ‐RIP3/MLKL necroptosis [33]. This pathological mitophagy degrades MFN2 and activates necroptosis via the MAM‐CaMKIIδ‐RIP3/MLKL axis, linking mitochondrial‐ER crosstalk to cell death. Moreover, lipopolysaccharide‐induced citrate accumulation in lung injury binds to FUNDC1, synergizing mitochondrial fission with PINK1/Parkin hyperactivation to drive necroptosis [15]. These findings underscore the context‐dependent role of mitophagy in necroptosis (Figure 2).

Mitophagy's dual role hinges on ROS dynamics and mitochondrial network integrity. Physiological mitophagy buffers ROS to prevent necroptosis, whereas excessive mitophagy triggers cytotoxic ROS surges. MFN2 degradation impairs mitochondrial dynamics, sensitising cells to ER stress‐driven necroptosis. Direct links between PINK1/Parkin and necrosome components (e.g., RIPK1/MLKL transcriptional regulation) remain unknown. Future work should determine if mitophagy modulates necroptosis through organelle removal, metabolic reprogramming, or signalling to identify therapeutic avenues.

#### 2.2.2 Ub‐Independent Pathways

Ub‐independent mitophagy, mediated by OMM receptors, such as Nip3‐like protein X (NIX)/BNIP3L, BNIP3, and FUNDC1, bypasses ubiquitination by directly binding to LC3 through LC3‐interacting regions (LIRs) [15]. Although recent studies have suggested that this pathway modulates necroptosis, mechanistic insights remain limited. Genetic ablation of Nix and Bnip3 in murine IgG^+^ memory B cells elevated necroptotic markers (e.g., RIPK1/MLKL), implying that NIX/BNIP3L‐mediated mitophagy suppresses necroptosis by maintaining mitochondrial homeostasis [34]. Conversely, intestinal I/R injury induced neutrophil extracellular trap (NET) formation, which disrupted FUNDC1‐dependent mitophagy via dysregulated phosphorylation (increased at Tyr18, and decreased at Tyr17), exacerbating mitochondrial dysfunction and necroptosis‐driven intestinal damage [35]. These findings suggest that, in the studied models, Ub‐independent mitophagy may act as a protective mechanism against necroptosis. However, whether receptor‐specific mitophagy exhibits context‐dependent effects (e.g., hypoxia and drug stimuli) akin to PINK1/Parkin and how mitochondrial dynamics (e.g., fission/fusion) or metabolic shifts interface with necrosome activation remain unknown. A systematic investigation of disease‐specific models is required to uncover therapeutic targets at the mitophagy‐necroptosis nexus (Figure 2).

### 2.3 Mitochondrial Biogenesis

Mitochondrial biogenesis, the expansion and replication of pre‐existing mitochondria, is a tightly regulated process that is essential for maintaining cellular energy homeostasis [36]. This requires the coordinated synthesis of mitochondrial membranes, replication of mitochondrial DNA (mtDNA), and import of nuclear‐encoded mitochondrial proteins [37]. PGC‐1α, a master regulator of mitochondrial biogenesis, drives mitochondrial mass expansion and oxidative metabolism through coordinated activation of nuclear and mitochondrial genes. Additionally, PGC‐1α enables adaptation to energy demands by upregulating electron transport chain components and metabolic enzymes while integrating mitochondrial function with pathways such as fatty acid oxidation and thermogenesis.

Emerging evidence suggests an apparent negative correlation between mitochondrial biogenesis and necroptosis activation in disease contexts, although mechanistic causality remains elusive. In this regard, doxorubicin‐induced cardiotoxicity, characterized by oxidative stress, cardiac fibrosis, and necroptosis marker upregulation (e.g., RIPK1/3), was partially mitigated by the PGC‐1α agonist LZN005, which reduced lipid peroxidation and necroptotic signalling [38]. Similarly, aging murine hearts and acute kidney injury models exhibited concurrent suppression of PGC‐1α and elevation of necroptotic effectors (RIPK3, MLKL) [39, 40]. Conversely, enhanced mitochondrial biogenesis coupled with necroptosis inhibition improved cell viability in cytomegalovirus‐infected cardiomyocytes [41]. These observations imply a compensatory interplay, wherein PGC‐1α‐driven mitochondrial biogenesis may counterbalance necroptotic cell death under stress, potentially by alleviating metabolic dysfunction or oxidative damage.

Current evidence remains correlative, lacking direct validation of whether PGC‐1α regulates necroptosis through transcriptional control of necrosome components or via indirect mechanisms (e.g., ROS scavenging, metabolic reprogramming). Targeted studies—such as those using tissue‐specific PGC‐1α knockout models combined with necroptosis inhibitors—are required to elucidate this causal interplay and exploit this axis for therapeutic benefit in conditions such as cardiomyopathy or sepsis‐induced organ failure.

### 2.4 Mitochondrial Protein Homeostasis

The mitochondrial proteome is primarily encoded by the nucleus, with only a small fraction synthesized internally. Nuclear‐derived proteins enter the mitochondria as unfolded precursors, a process that is vulnerable to misfolding caused by oxidative stress or chaperone dysfunction. Mitochondria utilize ATP‐dependent proteases, such as Lon protease 1 (LONP1), caseinolytic protease (ClpP), and m‐AAA/i‐AAA, to counteract proteostatic collapse, which degrade misfolded proteins via ATP hydrolysis and preserve mitochondrial integrity. For instance, LONP1 mutations disrupt the mitochondrial surveillance mechanism, causing cerebral, ocular, dental, auricular, and skeletal syndromes, multisystem disorders marked by developmental anomalies and mitochondrial dysfunction [42]. These findings underscore the non‐redundant role of protein quality control in counteracting proteotoxicity, which, if unaddressed, may propagate stress signals to cell death pathways.

Recent studies have indicated that mitochondrial protease dysfunction can act as a trigger for necroptosis. Murru et al. demonstrated that astrocyte‐specific deletion of Afg3l1 and Afg3l2 (subunits of the m‐AAA protease) in mice induced mitochondrial fragmentation, cristae disassembly, and necroptosis activation [43]. This was evidenced by upregulated expression of RIPK3 and Z‐DNA‐binding protein 1 (Zbp1) expression, key mediators of the necroptotic cascade. The results suggested that m‐AAA protease loss compromises the clearance of damaged mitochondrial proteins, leading to proteotoxic stress that converges with RIPK3‐driven signalling. Critical gaps in the literature persist: whether astrocyte necroptosis directly contributes to neurodegeneration or is merely a bystander effect remains unproven; the mechanistic crosstalk between other proteases (e.g., ClpP and i‐AAA) and necrosome components (MLKL and PGAM5) is undefined; and current evidence relies heavily on genetic models, neglecting the pharmacological modulation of protein quality control as a therapeutic strategy. Addressing these questions will help elucidate how mitochondrial proteostasis interfaces with necroptotic pathways, thereby offering novel targets for diseases ranging from brain disorders to ischemic injuries.

## 3 Mitochondrial Energy Metabolism and Necroptosis

The association between mitochondrial energy metabolism and necroptosis has been studied since the 1990s. Fiers et al. first revealed that mitochondrial complex I‐generated ROS are critical for TNF‐induced necrosis in L929 cells [44]. This discovery established ROS as a key player in necroptosis. Subsequently, Peter et al. identified RIP3 as a central regulator and showed that the activation of metabolic enzymes (PYGL, GLUL, and GLUD1) drives bioenergetic shifts and sustains ROS production [4]. Thus, ROS act as by‐products of disrupted metabolism and directly trigger necroptosis, creating a self‐reinforcing cycle that worsens mitochondrial dysfunction. These findings highlight ROS as pivotal mediators connecting mitochondrial energy imbalances to necroptotic cell death, thereby emphasizing the metabolic basis of this process.

### 3.1 Glycolysis and ROS: Amplifiers of Necroptosis

The interplay between glucose metabolism and necroptosis is mediated by ROS, which act as initiators and amplifiers of the cell death cascade. ROS generation, primarily originating from mitochondrial OXPHOS and NADPH oxidase systems, is tightly regulated by glycolytic activity [45, 46]. However, necroptotic stimuli drive glycolytic upregulation to fuel ROS production, creating a self‐perpetuating cycle that exacerbates cellular damage [4]. The addition of glycolysis inhibitors to U937 and Jurkat cells attenuates necroptosis by disrupting ROS production [47]. This regulatory axis is further exemplified in *Staphylococcus aureus* ‐infected keratinocytes, where pathogen‐induced glycolytic flux enhances tricarboxylic acid cycle activity and mtROS production, culminating in mitochondrial membrane depolarization and necroptosis [48]. Although these findings underscore glycolysis as a metabolic rheostat for ROS‐driven necroptosis, the precise molecular crosstalk remains unclear.

### 3.2 Pyruvate Metabolism: Balancing ROS and Necroptosis

Pyruvate, the terminal product of glycolysis, serves as a metabolic nexus linking mitochondrial bioenergetics to redox balance [49]. Following mitochondrial import via the pyruvate dehydrogenase complex (PDC), pyruvate fuels the tricarboxylic acid cycle and acts as a direct ROS scavenger through non‐enzymatic decarboxylation [50]. Huang et al. demonstrated that exogenous pyruvate derivatives mitigated necroptosis in hypoxic colorectal cancer cells by suppressing mtROS accumulation, RIP1/RIP3 necrosome assembly, and lactate dehydrogenase‐mediated membrane damage [51]. Paradoxically, pyruvate kinase M2 (PKM2), a key glycolytic enzyme that catalyses pyruvate synthesis, promotes necroptosis during cisplatin‐induced acute kidney injury. Upregulation of renal tubular PKM2 drives Drp1‐dependent mitochondrial fragmentation and necroptotic protein activation, as evidenced by reduced cell death in PKM2‐knockout mice and PKM2 inhibitor‐treated models [52]. Further complexity arises from RIP3‐MLKL necrosome translocation to the mitochondria, where PDC‐E3 subunit phosphorylation enhances aerobic respiration and mtROS production, establishing a feed‐forward loop that reinforces necroptotic signalling [53]. These dual roles highlight pyruvate metabolism as a context‐dependent modulator of necroptosis.

### 3.3 Hexokinase‐II (HK‐II) Governs Metabolic Control of Necroptosis

HK‐II, a hypoxia‐inducible isoform that anchors glycolysis to mitochondrial membranes, exerts paradoxical effects on necroptosis [54]. While HK‐II overexpression typically suppresses ROS production and cell death, its inhibition activates necroptotic pathways in cancer models. Uludağ et al. reported that the HK‐II inhibitor C‐10 induces necroptosis in glioblastoma cells, underscoring its therapeutic potential [55]. Similarly, sulconazole‐mediated HK‐II downregulation in oesophageal cancer elevates intracellular ROS levels and necroptotic incidence, revealing that HK‐II is a metabolic checkpoint that restrains cell death [56]. These findings suggest that HK‐II is a dynamic regulator of necroptosis, with its pro‐survival or pro‐death roles contingent on the cellular context and metabolic demands.

### 3.4 OXPHOS Drives Necroptosis via Respiratory Complexes

Mitochondrial respiratory chain complexes are central to necroptosis because of their dual roles in ATP synthesis and ROS generation. Pharmacological inhibition of necroptosis rescues mitochondrial respiration, linking necroptotic suppression to OXPHOS preservation [57]. Sun et al. demonstrated that 2‐methoxy‐6‐acetyl‐7‐methylbutanedione treatment in colon cancer cells induces RIP1/RIP3 autophosphorylation and necrosome formation. These molecular complexes trigger calcium overload and JNK activation, which promote mitochondrial dysfunction through excessive ROS production from Complex II (succinate dehydrogenase), ultimately driving necroptosis [58]. Similarly, metformin/efavirenz/fluoxetine co‐treatment amplified ROS through Complex I/III inhibition, triggering DNA damage and necroptotic cell death [59]. In contrast, Liu et al. demonstrated that the Hippo effector TEAD1/YAP1 maintains mitochondrial function in murine cardiomyocytes [60]. Tead1 deletion impairs mitochondrial Complex I (NADH dehydrogenase) activity, causing ATP depletion and ROS accumulation, which drive cardiomyocyte necroptosis. This mitochondrial failure precipitates fatal acute dilated cardiomyopathy in mice. Although respiratory chain blockade generally attenuates necroptosis by curbing ROS, Complex II has emerged as a paradoxical source of ROS, warranting further exploration of its context‐specific roles.

## 4 THE mPTP AND NECROPTOSIS

The mPTP, a regulated non‐selective channel spanning the contact sites between the IMM and OMM, serves as a critical mediator of mitochondrial dysfunction. mPTP opening triggers rapid mitochondrial depolarization and initiates cellular death. Despite decades of investigation, the molecular identity of the mPTP remains controversial. Early models posited that physiological interactions between voltage‐dependent anion channels (VDAC) and adenine nucleotide translocases (ANT) undergo structural reorganization to form pathological mPTP [61]. Subsequent studies have proposed a bidirectional regulatory paradigm wherein strong stimuli induce unregulated pore formation, whereas weaker signals activate transient regulated openings [62]. Recent advances have expanded this framework through three competing hypotheses: the F1F0‐ATP synthase dimer model, the C‐ring subunit hypothesis and the multi‐component assembly theory [63, 64, 65]. Mechanistically, mPTP activation exhibits calcium dependency and cyclophilin D (CypD)‐mediated potentiation. Sustained pore opening precipitates catastrophic mitochondrial swelling, OMM rupture, and release of pro‐death factors, thereby bridging apoptotic and necrotic pathways [66]. This dual capacity positions the mPTP as a metabolic sentinel that determines cellular fate under stress.

### 4.1 CypD‐mPTP Axis: Key Regulator of Necroptosis

Mitochondrial CypD, the only genetically confirmed core regulator of the mPTP, is translocated to the IMM through oxidative stress and other stimuli and directly mediates mPTP opening and triggers mitochondrial depolarization, which is generally considered to be the initial event of necroptosis [67]. Chen et al. found that glial cells exhibited time‐dependent mPTP opening and necroptosis in response to mechanical compression and that cyclosporine A inhibition of CypD significantly attenuated cell death, corroborating the upstream regulation of the CypD‐mPTP axis [68]. Additionally, a study reported that CypD drives the continuous opening of the mPTP in an oxalate‐induced renal tubular injury model, leading to OMM permeability, crista structure disintegration, and ROS burst, which promotes the assembly of RIPK1/RIPK3/MLKL necrosomes, forming a positive feedback loop of mPTP open‐ROS‐necrosomes [69]. CypD‐mediated mPTP opening triggered mitochondrial membrane potential collapse and promoted necrosome formation via ROS production in cisplatin‐treated L929 cells, suggesting that this pathway has a bidirectional regulatory role in chemotherapy‐related necroptosis [70].

**FIGURE 3:**
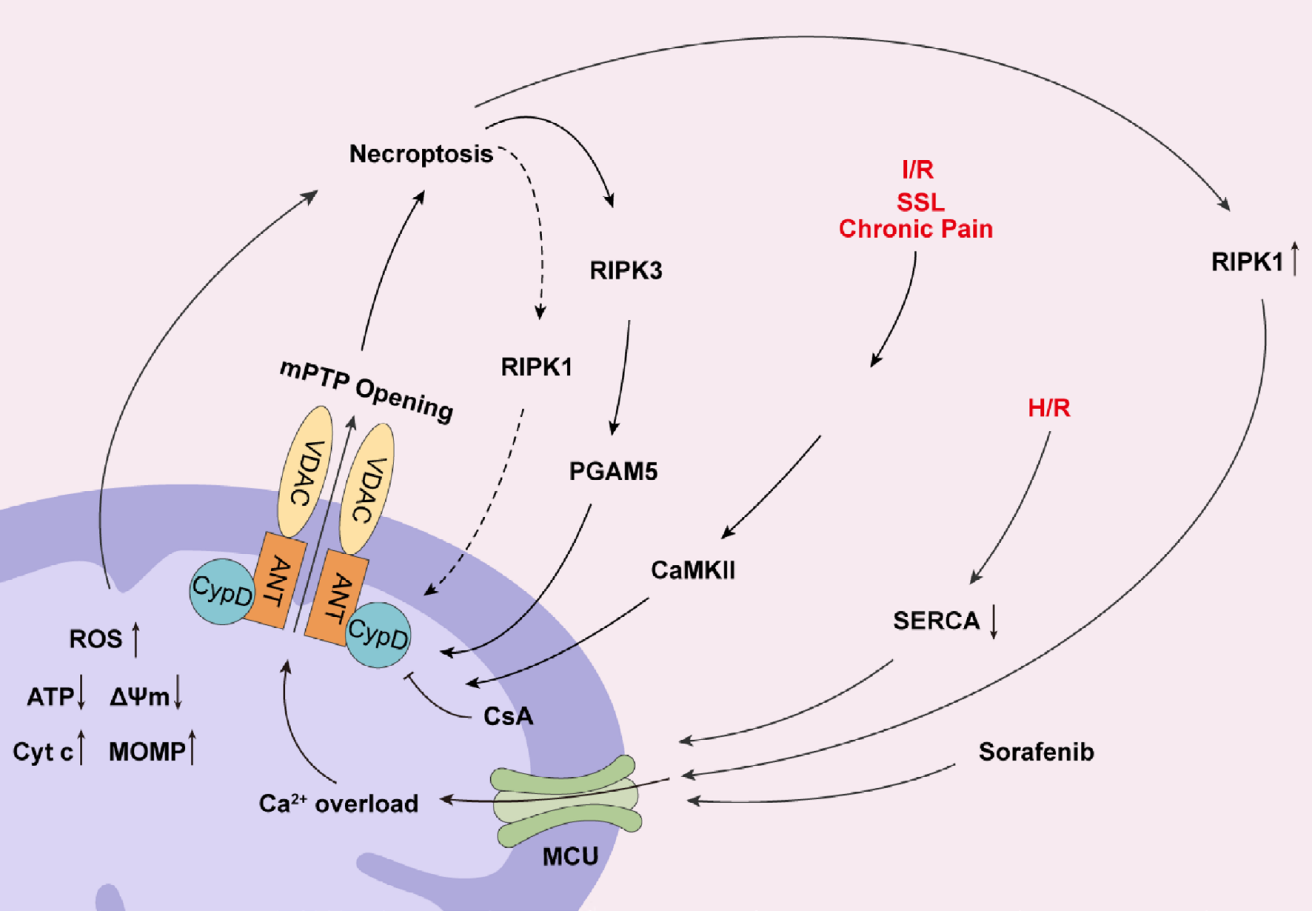
Mechanism of necroptosis caused by dysfunctional mitochondrial permeability transition pore. In the CypD‐mPTP axis, CypD directly mediates mPTP opening in response to stimuli such as inhibition by cyclosporine A (CsA), whereas upregulation of PGAM5 by an upstream kinase enhances CypD phosphorylation and prolongs mPTP opening, thereby triggering the necrosis pathway. The oligomerization of VDAC1 and ANT in the VDAC1‐mPTP nexus, as the core structural components of mPTP, leads to excessive activation of mPTP under hypoxia/reoxygenation or I/R stimulation, resulting in Ca^2+^ overload and mitochondrial dysfunction. However, the role of VDAC1 is contradictory, and knockout of VDAC1 may enhance necroptosis. MCU regulates Ca^2+^ uptake, and Ca^2+^ overload induced by sarco/endoplasmic reticulum Ca^2+^‐ATPase or sorafenib promotes mPTP opening. Meanwhile, CaMKII phosphorylation by RIPK3 (upon I/R, staphylococcal‐like superantigen, or chronic pain stimulation) independently induces mPTP opening, forming a necrotic execution pathway parallel to MLKL.

The abnormal opening of the mPTP has been shown to be a key driver of necroptosis in cardiac I/R injury [71, 72]. RIPK3 enhances CypD phosphorylation by upregulating PGAM5, which prolongs the mPTP opening time and exacerbates endothelial cell necroptosis, thus establishing PGAM5 as a positive regulator of the mPTP [73]. I/R induced ROS overproduction through the ER‐Ca^2+^‐xanthine oxidase signalling axis, which in turn activated the mPTP and initiated the necroptotic program; however, RIPK3 gene knockdown blocked this pathway [74]. Although existing evidence indicates that RIPK3 is a core regulatory mediator upstream of mPTP, recent studies have suggested that RIPK1 may also regulate mPTP activity through a pathway independent of RIPK3 [75]. These findings suggest that the necroptosis‐associated kinases, RIPK1/RIPK3, dynamically control the opening threshold of mPTP in response to pathological stress by modifying CypD or regulating its microenvironment, thereby determining the necroptotic fate of cells (Figure 3).

### 4.2 The VDAC1‐mPTP Nexus: Dual Faces in Necroptotic Regulation

The ANT/VDAC/CypD model was predominant in mPTP research, positing ANT and VDAC as core structural components, with CypD and auxiliary proteins such as Bax modulating pore activation [76]. Additionally, VDAC1, the most abundant OMM protein, was established as a critical metabolic gatekeeper, facilitating the bidirectional transport of metabolites (< 5 kDa) and serving as a Parkin substrate for mitophagy regulation [77, 78]. Although mammals express three VDAC isoforms, early investigations predominantly focused on VDAC1 because of its centrality in cellular bioenergetics [79]. Recent studies have explored the role of VDAC1 in necroptosis. Hypoxia‐induced lncRNA HABON silencing in hepatoma Huh‐7 cells triggered VDAC1‐dependent mPTP opening, manifesting as ATP depletion, mtROS surge, and mitochondrial depolarization, events culminating in necroptosis [80]. Similarly, Wan et al. linked OGD/R‐induced VDAC1 oligomerization in retinal neurons to mPTP hyperactivation, cytosolic Ca^2+^ overload, and cytochrome c release, ultimately driving necroptosis demise [81]. These findings implicate VDAC1 as an mPTP modulator that bridges metabolic stress and necroptotic execution.

However, Zhang et al. reported that VDAC1 knockdown in breast cancer cells paradoxically enhanced TSZ‐induced necroptosis, contradicting the proposed pro‐necroptotic function [82]. This discrepancy suggests context‐dependent roles for VDAC1 that are potentially mediated by isoform‐specific interactions or compensatory mechanisms within the VDAC family [83]. Although the evidence implicates VDAC oligomerization as a determinant of mPTP permeability, rigorous validation of VDAC isoforms during necroptosis remains scarce. No study has conclusively delineated whether VDAC2 or VDAC3 compensates for VDAC1 deficiency in mPTP regulation. These gaps underscore the need to reevaluate the historical ANT/VDAC/CypD framework through isoform redundancy and tissue‐specific mPTP composition (Figure 3).

### 4.3 Calcium Crossroads: Mitochondrial Calcium Uniporter (MCU) and Calcium/Calmodulin‐Dependent Protein Kinase II (CaMKII) in mPTP‐Driven Necroptosis

Mitochondrial Ca^2+^ dynamics, governed by the MCU, critically regulate mPTP opening—a process potentiated by Ca^2+^ overload [84]. While physiological MCU activity sustains aerobic metabolism and survival, pathological hyperactivation drives necroptosis via mPTP‐dependent cascades. RIPK1 upregulation in colorectal cancer enhanced MCU ubiquitination at K377, boosting mitochondrial Ca^2+^ uptake to fuel tumour proliferation [85]. Conversely, Song et al. revealed that sorafenib‐induced MCU overactivation in cardiomyocytes triggers Ca^2+^ overload, RIPK3/MLKL phosphorylation, and necroptosis [32]. This dual role is further exemplified in cardiac microvascular endothelial cells, where hypoxia/reoxygenation downregulates sarco/endoplasmic reticulum Ca^2+^‐ATPase, causing cytosolic Ca^2+^ influx into the mitochondria via the MCU, mPTP opening, and necroptotic cell death [86]. Paradoxically, Parks et al. observed that MCU‐knockout mice lacked cardiac protection due to compensatory CypD phosphorylation at S42, which lowered the Ca^2+^ sensitivity of mPTP, enabling pore activation independent of matrix Ca^2+^ [87]. These findings underscore the context‐dependent outcomes of MCU modulation, where compensatory mechanisms may bypass the canonical Ca^2+^‐mPTP coupling.

Although MLKL is indispensable for necroptosis, its inhibition fails to fully abrogate cardiomyocyte death, suggesting the involvement of alternative pathways [88]. CaMKII has recently emerged as a RIPK3 substrate that regulates mPTP‐driven necroptosis. I/R stress upregulates RIPK3, which phosphorylates CaMKII to induce mitochondrial depolarization and mPTP opening by bypassing MLKL [89]. Yang et al. expanded this paradigm in myocardial I/R models, showing that chronic pain amplifies the RIPK3‐MLKL and RIPK3‐CaMKII axes and that melatonin pretreatment attenuates necroptosis [90]. Furthermore, staphylococcal‐like superantigen toxins were found to bind TNFR1 to activate RIPK3‐CaMKII‐mPTP signalling, directly linking pathogen recognition to mitochondrial permeability shifts [91]. These studies redefine necroptosis as a multiplex signalling network, wherein CaMKII serves as a parallel executor to MLKL, particularly under conditions of metabolic or infectious stress (Figure 3).

## 5 Mitochondrial Solute Carriers and Necroptosis

Emerging evidence suggests that mitochondrial solute carriers (SLCs) are critical modulators of necroptosis, although their mechanistic roles remain unclear. A seminal study demonstrated that genetic ablation of the zinc transporter SLC39A7 confers resistance to necroptosis in FADD‐deficient haploid human KBM7 cells treated with TNF‐α or SMAC mimetics [92]. This resistance was mechanistically linked to the SLC39A7‐dependent regulation of TNFR expression, suggesting a novel role for mitochondrial zinc homeostasis in necroptotic signalling. In contrast, Ma et al. identified SLC10A3 as a potential promoter of necroptosis and tumour immunosuppression in low‐grade gliomas [93]. Elevated SLC10A3 expression was correlated with enhanced necroptotic activity, immunosuppressive tumour microenvironment remodelling, and poor clinical prognosis in patients with low‐grade gliomas. These findings highlight the dual regulatory roles of SLCs in suppressing or amplifying necroptosis, depending on their substrate specificity and cellular context. Nevertheless, the causal relationships remain unclear. For instance, the involvement of SLC10A3 in necroptosis is currently supported only by correlative data, necessitating functional validation. Consequently, further research should delineate how SLC‐mediated metabolite transport integrates with canonical necroptotic pathways (e.g., the RIPK1/RIPK3/MLKL axis) and explore the therapeutic targeting of SLCs to modulate mitochondria‐driven cell death in diseases such as cancer and neurodegeneration.

## 6 Perspectives

In this review, we elucidated the role of mitochondrial dysfunction in the development of necroptosis and summarized the key proteins and processes involved. We found that different or even diametrically opposing results have been produced under different stimulus conditions and disease models. Additionally, detailed mechanistic investigations remain limited. Moreover, effective clinical evidence for the intervention of key proteins remains inadequate, leaving considerable scope for therapeutic translation. Consequently, future studies should elucidate the mechanisms of action of the key proteins that impact mitochondrial function during necroptosis and determine whether it is specific to different types of disease models. Understanding the intrinsic mechanisms by which the key proteins that cause mitochondrial dysfunction contribute to necroptosis in different disease models will help explore the potential of targeted blockade or induction of mitochondrial proteins as novel therapeutic agents for various diseases.

## Author Contributions

**Haiying Rui:** conceptualization, writing – review and editing. **Li Xue:** conceptualization, writing – review and editing. **Dandan Qin:** writing – review and editing. **Yuguo Chen:** conceptualization. **Huaxiang Yu:** writing – review and editing. **Yuting Li:** writing – review and editing. **Fengxin Wang:** conceptualization, writing – original draft, writing – review and editing. **Dan Zou:** writing – review and editing. **Wenyi Zou:** writing – review and editing. **Ran Li:** writing – review and editing.

## Funding

This study was supported by the National Natural Science Foundation of China (82030059, U23A20485 to author YC, 81300219, 81671951 to author LX), National Key R&D Program of China (2020YFC1512700 to author YC, 2020YFC1512705 to author YC, 2020YFC1512703 to author YC), Natural Science Foundation of Shandong Province (ZR2013HQ058 to author LX, ZR2022MH078 to author LX).

## Ethics Statement

The authors have nothing to report.

## Consent

The authors have nothing to report.

## Conflicts of Interest

The authors declare no conflicts of interest.

## Data Availability

Data sharing not applicable to this article as no datasets were generated or analysed during the current study.
